# Popliteal cysts and subgastrocnemius bursitis are associated with knee symptoms and structural abnormalities in older adults: a cross-sectional study

**DOI:** 10.1186/ar4496

**Published:** 2014-03-03

**Authors:** Yuelong Cao, Graeme Jones, Weiyu Han, Benny Antony, Xia Wang, Flavia Cicuttini, Changhai Ding

**Affiliations:** 1 Menzies Research Institute Tasmania, University of Tasmania, Private Bag 23, Hobart, Tasmania 7000, Australia; 2 Research Institute of Orthopaedics, Shuguang Hospital Affiliated to Shanghai University of Traditional Chinese Medicine, 528 Zhangheng Road, Shanghai 201203, China; 3 Department of Epidemiology and Preventive Medicine, Monash University, Melbourne 3004, Australia

## Abstract

**Introduction:**

The role of popliteal cysts and subgastrocnemius bursitis in knee joint homeostasis is uncertain. The aim of this study is to describe cross-sectional associations between popliteal cysts, subgastrocnemius bursitis, knee symptoms and structural abnormalities in older adults.

**Methods:**

A cross-sectional sample of 900 randomly-selected subjects (mean age 63 years, 48% female) were studied. Knee pain, stiffness and dysfunction were assessed by self-administered Western Ontario McMaster Osteoarthritis Index (WOMAC) questionnaire. Radiographic knee osteophyte and joint space narrowing (JSN) were recorded. Magnetic resonance imaging (MRI) was utilized to assess popliteal cysts, subgastrocnemius bursitis, cartilage defects and bone marrow lesions (BMLs).

**Results:**

Popliteal cysts were present in 11.7% and subgastrocnemius bursitis in 12.7% of subjects. Subgastrocnemius bursitis was more common in those with popliteal cyst (36.2% versus 9.7%, *P* <0.01). In multivariable analyses, popliteal cysts were significantly associated with increased osteophytes in both medial and lateral tibiofemoral compartments while subgastrocnemius bursitis was associated with increased osteophytes and JSN in the medial tibiofemoral compartment. Both were significantly associated with cartilage defects in all compartments, and with BMLs in the medial tibiofemoral compartment. Furthermore, both popliteal cysts and subgastrocnemius bursitis were significantly associated with increased weight-bearing knee pain but these associations became non-significant after adjustment for cartilage defects and BMLs.

**Conclusions:**

Popliteal cysts and subgastrocnemius bursitis are associated with increased symptoms as well as radiographic and MRI-detected joint structural abnormalities. Longitudinal data will help resolve if they are a consequence or a cause of knee joint abnormalities.

## Introduction

Osteoarthritis (OA) is a slowly progressive and multifactorial disease characterised by gradual loss of articular cartilage and other structural changes in the whole joint [1]. Among these structural changes, cystic lesions in the knee joint are a common feature seen on magnetic resonance imaging (MRI) or ultrasound assessment but may not be detected by physical examination [2].

Most cystic lesions around the knee joint represent encapsulated fluid collections. Although bursae are synovium-lined structures and normally quiescent, bursitis can be detected when bursae become inflamed and/or filled with fluid [3]. Among those cystic lesions, popliteal cyst (Baker cyst [4]) and subgastrocnemius bursitis are most commonly detected on MRI with the prevalence of about 38% for popliteal cyst [5] and 15% [6] for subgastrocnemius bursitis. Popliteal cysts are usually formed from extrusion of joint fluid into the gastrocnemius-semimembranosus bursa [7] and can extend deep to gastrocnemius muscles, resulting in fluid in the subgastrocnemius bursa. A narrow neck connecting the bursa to the knee joint is usually identified on axial MRI images and is commonly found just below the proximal attachment site of the medial head of gastrocnemius [6]. Therefore, subgastrocnemius bursae can communicate with the knee joint and popliteal cyst if co-existing.

In adults, almost all popliteal cysts are secondary [8] since a communication exists between the cysts and the knee joint, allowing articular fluid to enter the cyst. It is therefore reasonable to postulate that popliteal cyst and subgastocnemius bursitis may be associated with intra-articular derangement of the knee joint; however, this has only been examined in a limited number of studies. Although popliteal cyst has been associated with the presence of a meniscal lesion, [5,9] it is unclear if popliteal cyst and subgastrocnemius bursitis play roles in radiographic OA, and MRI-detected structural abnormalities. The aim of our study was, therefore, to describe the prevalence of popliteal cyst and subgastrocnemius bursitis in a cohort of older adults and to examine their associations with radiographic and MRI-detected structural abnormalities, as well as knee symptoms.

## Methods

### Study design and subjects

The study was based on the Tasmanian Older Adult Cohort (TASOAC) study, a prospective epidemiological study of persons aged 50 to 79 years, with a goal of identifying the environmental, genetic and biochemical factors associated with the development and progression of OA and osteoporosis. Participants were selected randomly using computer-generated random numbers from the electoral roll in Southern Tasmania (population 229,000), a comprehensive population listing, with an equal number of men and women. Institutionalised persons were excluded. The study was approved by the Southern Tasmanian Health and Medical Human Research Ethics Committee, and written informed consent was obtained from all participants. Self-reports of disease status such as rheumatoid arthritis (RA), asthma, cardiovascular disease and diabetes were recorded by questionnaire. Baseline measurements were conducted from April 2002 to September 2004, and the overall response rate was 57%.

### Anthropometrics

Height was measured to the nearest 0.1 cm by using a stadiometer, with shoes, socks and headgear removed. Weight was measured to the nearest 0.1 kg (with shoes, socks and bulky clothing removed) using a single pair of electronic scales (Seca Delta Model 707, Bradford, MA, USA) that were calibrated using a known weight at the beginning of each clinic. Body mass index (BMI) (weight (kg)/height^2^ (m^2^)) was calculated.

### MRI

Using a commercial transmit/receive extremity coil, MRI of the right knee was performed with a 1.5 T whole-body magnetic resonance unit (Picker, Cleveland, OH, USA). The following sequence and parameters were used: a T1-weighted fat-suppressed three-dimensional gradient-recalled acquisition in the steady state, flip angle 30°, repetition time 31 ms, echo time 6.71 ms, field of view 16 cm, 60 partitions, 512 × 512-pixel matrix, acquisition time 5 minutes 58 seconds, 1 acquisition, sagittal images obtained at a partition thickness of 1.5 mm without a between-slice gap; and a T2-weighted fat-saturated two-dimensional fast spin echo, flip angle 90°, repetition time 3,067 ms, echo time 112 ms, field of view 16 cm/15 partitions, 228 × 256-pixel matrix; sagittal images were obtained at a partition thickness of 4 mm with a between-slices gap of 0.5 to 1.0 mm. Assessments were performed on a standard digital imaging and communications in medicine (DICOM) viewer (Osiris 4, University of Genevia, Geneva, Switzerland).

### Popliteal cyst and subgastrocnemius bursitis assessments

**Figure 1:**
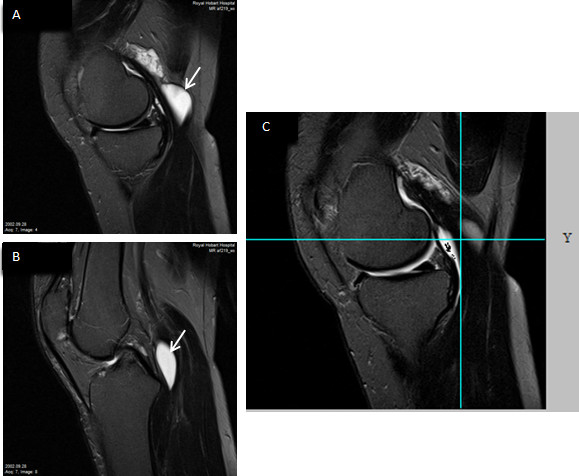
**Simultaneous presence of a popliteal cyst (Baker cyst) and subgastrocnemius bursitis in a right knee.** A grade-3 popliteal cyst (arrow) was superficially located adjacent to the medial head tendon of the gastrocnemius **(A)**; also a grade-3 subgastrocnemius bursitis (arrow) was shown between the capsule and medial head of the gastrocnemius **(B)**; the popliteal cyst and subgastrocnemius bursitis were separated but communicated with each other in the multiplanar view in the sagittal plane **(C)**.

Assessments were performed on T2-weighted sagittal and axial views (Figure 1). Following to a previously published method, [10,11] popliteal cysts and subgastrocnemius bursitis were scored using a semiquantitative scale: grade 0 for absent, grade 1 for small, grade 2 for moderate, grade 3 for large. Interobserver reproducibility was assessed between two readings in 82 images of randomly selected subjects, with interclass correlation coefficients of 0.87 for subgastrocnemius bursitis and 0.86 for popliteal cysts. A further random subset of these images was re-read for intraclass correlation coefficients (ICCs) with 0.93 and 0.94 for subgastrocnemius bursitis and popliteal cysts, respectively.

### Cartilage defects and bone marrow lesion (BML) assessments

Cartilage defects were assessed at the medial tibial, medial femoral, lateral tibial, and lateral femoral sites as previously reported [12]: grade 0 = normal cartilage; grade 1 = focal blistering and intracartilaginous low-signal intensity area with an intact surface and bottom; grade 2 = irregularities on the surface or bottom and loss of thickness <50%; grade 3 = deep ulceration with loss of thickness >50%; grade 4 = full-thickness chondral wear with exposure of subchondral bone. A cartilage defect also had to be present in at least two consecutive slices. ICCs were 0.89 to 0.94, and interclass correlation coefficients were 0.85 to 0.93 [13].

BMLs were assessed on the T2-weighted images and defined as discrete areas of increased signal adjacent to the subcortical bone at the medial tibial, medial femoral, lateral tibial, and lateral femoral sites as previously described [14]. Each BML was scored on the basis of lesion size: grade 0 = no BML; grade 1 = only present on one slice; grade 2 = on two consecutive slices; grade 3 = on more than three consecutive slices. The highest score was used if more than one lesion was present on the same site. Intraobserver repeatability was assessed in 50 subjects with at least a one-week interval between two readings, with ICCs of 0.89, 0.96, 0.94, and 1.00 for the BML scores at lateral tibia, lateral femur, medial tibia, and medial femur, respectively.

Knee effusion was graded from 0 to 3 in terms of the estimated maximal distension according to the whole-organ magnetic resonance imaging score (WORMs) method [11].

### Western Ontario McMaster osteoarthritis index (WOMAC) and knee pain assessment

Each component of knee pain, stiffness and dysfunction was assessed by self-administered WOMAC questionnaire [15] with a10-point scale from 0 (no symptom) to 9 (most severe symptom). Total knee pain was the sum of all components to create a total pain (0 to 45) score. The five WOMAC pain questions related to knee OA were clinically constructed in three weight-bearing pain questions (pain on climbing stairs, on walking, and on standing) and two non-weight-bearing pain questions (pain in bed or when sitting/lying down) as suggested by a recent study [16].

### Radiographic OA assessment

This was scored according to the Osteoarthritis Research Society International (OARSI) atlas [17] as previously reported [13]. A standing semi-flexed view of the right knee with 15º of fixed knee flexion was performed in all subjects and scored individually for joint space narrowing (JSN) and osteophytes on a scale of 0 to 3 (0 = normal and 3 = severe). Medial or lateral JSN was scored separately, and osteophytes were scored at each site of the medal tibia, medial femur, lateral tibia and lateral femur.

### Data analysis

The Student *t*-test or χ^2^ test was used to compare means or proportions, respectively, between those with and without popliteal cyst. Ordinal logistic regression was used to examine the associations between popliteal cysts, subgastrocnemius bursitis (dependent variables) and the anthropometric/other risk factors (independent variables). Univariable and multivariable ordinal logistic regression analysis was used to examine the associations between dependent variables (score of knee cartilage defects, BMLs, osteophytes and JSN in the medial or lateral tibiofemoral site) and independent variables (each score for popliteal cyst and subgastrocnemius bursitis) before and after adjustment for age, sex, BMI, smoking status, radiographic features, and/or disease status (RA, cardiovascular disease, asthma and diabetes). Binary logistic regression models were used to examine the associations between the cystic lesion score (independent variables) and presence of knee symptoms, including weight-bearing pain, non-weight-bearing pain, total knee pain, stiffness and dysfunction (dependent variables). The associations between cystic lesions and knee symptoms were further adjusted for effusion or cartilage defects/BMLs to see if they were mediated by these structural abnormalities.

Standard diagnostic checks of model fit and residuals were routinely made, and data points with large residuals and/or high influence were investigated for data errors. Interactions between sex and cystic lesions on OA measures were investigated by testing the statistical significance of the coefficient of a product term (sex × a cystic lesion) after adjustment for confounders. A *P*-value of <0.05 (two-tailed) was regarded as statistically significant. All statistical analyses were performed on SPSS V.20.0 for Windows (SPSS, Chicago, IL, USA).

## Results

Of 900 subjects (49.7% female) included in the analysis, the average age at baseline was 62.3 years. The mean BMI was 27.7 kg/m^2^. Popliteal cysts were present in 105 (11.7%) subjects, with 47 in grade 1, 40 in grade 2, and 18 in grade 3. Subjects with or without popliteal cyst were similar in terms of age, gender, BMI, non-weight-bearing pain, total pain, prevalence of BMLs, JSN and effusion; however, subjects with popliteal cyst had more weight-bearing pain, and more osteophytes and cartilage defects at all sites (all P <0.05). Subgastrocnemius bursitis (prevalence 12.7%) was more common in those with popliteal cyst (36.2% versus 9.7%, *P* <0.01). Thus, 38 subjects had both popliteal cysts and subgastrocnemius bursitis, 67 had popliteal cyst and 77 had subgastrocnemius bursitis only (Table 1).

**Table 1:** Baseline characteristic of participants

**Characteristic**	**Without popliteal cyst (n = 795)**	**With popliteal cyst (n = 105)**	***P***
Age, years	62.2 (7.3)	63.2 (8.0)	0.18*
Female gender, %	49.9	47.6	0.65
Body mass index, kg/m^2^	27.6 (4.5)	27.9 (4.9)	0.53*
WOMAC knee pain			
Weight-bearing, 0 to 27 mm	**4.7 (4.9)**	**5.8 (5.0)**	0.03*
Non-weight-bearing, 0 to 18 mm)	3.2 (4.7)	3.1(4.7)	0.95*
Total pain, (0 to 45 mm	5.1 (5.0)	6.0 (4.9)	0.07*
Prevalence of bone marrow lesions, %	34.0	40.0	0.23
Prevalence of osteophytes, %	**8.2**	**16.2**	**0.01**
Medial tibiofemoral JSN, %	52.6	54.5	0.30
Lateral tibiofemoral JSN, %	23.4	27.3	0.34
Cartilage defect, ≥2, %			
Medial tibial	**20.4**	**80.4**	**<0.001**
Lateral tibial	**15.3**	**28.6**	**0.01**
Medial femoral	**19.7**	**31.4**	**<0.001**
Lateral femoral	**9.6**	**15.2**	**0.01**
Patellar	**38.9**	**49.5**	**0.01**
Effusion, WORMS ≥1, %	25.9	30.5	0.56
Subgastrocnemius bursitis, grade ≥1, %	**9.7**	**36.2**	**<0.001**

Results presented as mean (SD) except for percentages. Data in bold denote statistically significant results. JSN, joint space narrowing; WOMAC, Western Ontario McMaster Universities osteoarthritis index; WORMS, whole-organ magnetic resonance imaging score; *t-tests; others were χ^2^ tests.

Age, sex, BMI, and smoking were not associated with cystic lesions (data not shown); however, there was a significant association between history of knee surgery and the popliteal cyst score (knee surgery versus no knee surgery: β = 0.70, *P* = 0.01).

### Popliteal cyst, subgastrocnemius bursitis and radiographic features

**Figure 2:**
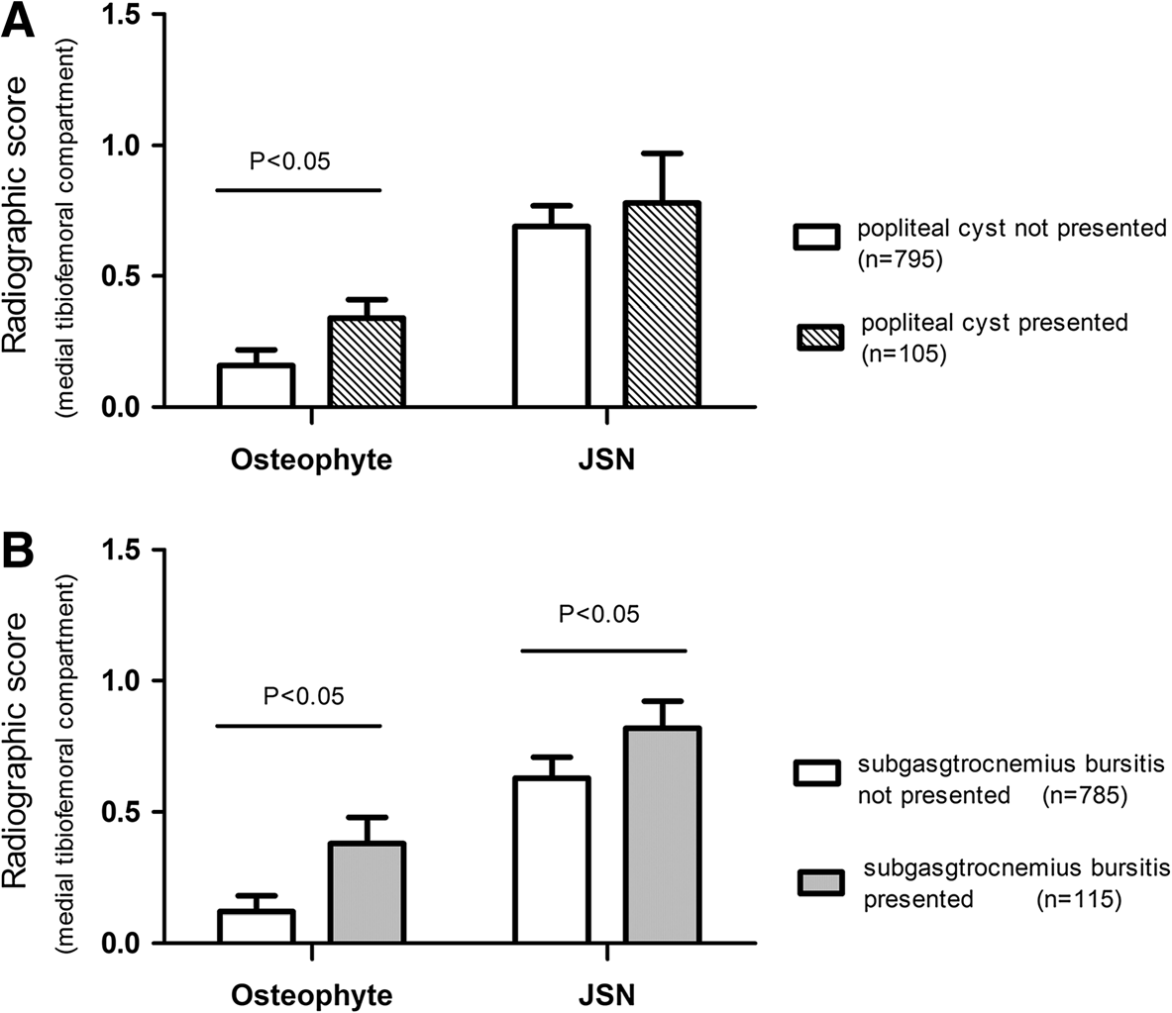
**Associations between popliteal cysts, subgastrocnemius bursitis, and knee radiographic features. (A)** Subjects with popliteal cysts had significantly higher medial tibiofemoral osteophyte score than those without popliteal cysts. **(B)** Subjects with subgastrocnemius bursitis had a significantly increased score for medial tibiofemoral joint space narrowing (JSN) and osteophytes compared with those without subgastrocnemius bursitis.

In multivariable analysis, whereas popliteal cysts were significantly associated with increased medial and lateral tibiofemoral osteophytes, subgastrocnemius bursitis was significantly associated with increased medial tibiofemoral JSN and osteophytes (Table 2, Figure 2). Popliteal cyst and subgastrocnemius bursitis were not significantly associated with lateral tibiofemoral JSN (Table 2).

**Table 2:** Association between subgastrocnemius bursitis, popliteal cyst and radiographic osteophytes or JSN

	**Univariable**			Multivariable*		
	**β**	**95% CI**	***P***	**β**	**95% CI**	***P***
Medial tibiofemoral JSN						
Subgastrocnemius bursitis	**0.32**	**(0.08, 0.56)**	**0.01**	**0.29**	**(0.05, 0.54)**	**0.01**
Popliteal cyst	0.04	(-0.17, 0.25)	0.68	-0.03	(- 0.25, 0.19)	0.77
Lateral tibiofemoral JSN						
Subgastrocnemius bursitis	-0.11	(-0.42, 0.19)	0.47	-0.17	(-0.49, 0.14)	0.28
Popliteal cyst	0.01	(-0.24, 0.26)	0.92	-0.02	(-0.28, 0.24)	0.87
Medial tibiofemoral osteophyte						
Subgastrocnemius bursitis	**0.63**	**(0.30, 0.95)**	**<0.001**	**0.61**	**(0.24, 0.97)**	**0.01**
Popliteal cyst	**0.48**	**(0.17, 0.78)**	**0.01**	**0.60**	**(0.25, 0.95)**	**0.01**
Lateral tibiofemoral osteophyte						
Subgastrocnemius bursitis	-0.02	(-0.67, 0.63)	0.95	-0.09	(-0.78, 0.58)	0.78
Popliteal cyst	**0.43**	**(0.03, 0.82)**	**0.03**	**0.46**	**(0.02, 0.91)**	**0.03**

Dependent variable: joint space narrowing (JSN) or osteophyte score. Independent variable: subgastrocnemius bursitis or popliteal cyst, per grade. *Adjusted for age, sex, body mass index, disease status, medial or lateral tibiofemoral osteophytes for JSN, or medial or lateral tibiofemoral JSN for osteophytes. Data in bold denote statistically significant results.

### Popliteal cyst, subgastrocnemius bursitis and MRI-detected structural features

Popliteal cyst or subgastrocnemius bursitis was significantly and positively associated with knee cartilage defects at medial and lateral tibofemoral sites before and after adjustment for age, sex, BMI, disease status and knee radiographic features. In univariable analysis both were also significantly associated with a patellar cartilage defect and remained significant, except for popliteal cyst, after adjustment for the covariates as mentioned above (Table 3). Moreover, in multivariable analysis both were significantly associated with medial tibiofemoral BMLs, but not with lateral tibiofemoral BMLs (Table 4).

**Table 3:** Association between subgastrocnemius bursitis, popliteal cyst and cartilage defects

	**Univariable**			Multivariable*		
	**β**	**95% CI**	***P***	**β**	**95% CI**	***P***
Medial tibiofemoral defects						
Subgastrocnemius bursitis	**0.41**	**(0.18, 0.64)**	**<0.001**	**0.40**	**(0.16, 0.65)**	**0.01**
Popliteal cyst	**0.39**	**(0.18, 0.59)**	**<0.001**	**0.32**	**(0.10, 0.53)**	**0.01**
Lateral tibofemoral defects						
Subgastrocnemius bursitis	**0.39**	**(0.16, 0.62)**	**0.01**	**0.38**	**(0.13, 0.62)**	**0.01**
Popliteal cyst	**0.47**	**(0.27, 0.67)**	**<0.001**	**0.41**	**(0.20, 0.62)**	**<0.001**
Patellar defects						
Subgastrocnemius bursitis	**0.37**	**(0.14, 0.59)**	**0.00**	**0.34**	**(0.10, 0.59)**	**0.01**
Popliteal cyst	**0.25**	**(0.04, 0.45)**	**0.01**	0.18	(-0.02, 0.39)	0.08

Dependent variable: cartilage defects. Independent variable: subgastrocnemius bursitis or popliteal cyst, per grade. *Adjusted for age, sex, body mass index, disease status, and radiographic osteoarthritis. Data in bold denote statistically significant results.

**Table 4:** Association between subgastrocnemius bursitis, popliteal cyst and BMLs

	**Univariable**			Multivariable*		
	**β**	**95% CI**	***P***	**β**	**95% CI**	***P***
Medial tibiofemoral BMLs						
Subgastrocnemius bursitis	0.20	(-0.06, 0.47)	0.13	**0.29**	**(0.01, 0.57)**	**0.03**
Popliteal cyst	**0.26**	**(0.03, 0.49)**	**0.02**	**0.26**	**(0.02, 0.49)**	**0.03**
Lateral tibofemoral BMLs						
Subgastrocnemius bursitis	0.20	(-0.07, 0.48)	0.15	0.18	(-0.12, 0.48)	0.24
Popliteal cyst	0.08	(-0.17, 0.34)	0.52	0.13	(-0.13, 0.39)	0.32

Dependent variable: bone mass lesions (BMLs). Independent variable: subgastrocnemius bursitis or popliteal cyst, per grade. *Adjusted for age, sex, body mass index, disease status, and radiographic osteoarthritis. Data in bold denote statistically significant results.

### Popliteal cyst, subgastrocnemius bursitis and knee symptoms

Both subgastrocnemius bursitis and popliteal cyst were associated significantly with the presence of weight-bearing knee pain, but not with the presence of non-weight-bearing pain or total knee pain, before and after adjustment for age, sex, BMI, disease status, and knee radiographic features. Moreover, subgastrocnemius bursitis was significantly associated with prevalent WOMAC stiffness and popliteal cyst was significantly associated with prevalent WOMAC dysfunction (Table 5). All these significant associations remained unchanged after further adjustment for effusion, but decreased in magnitude and became non-significant after further adjustment for cartilage defects and BMLs (Table 5).

**Table 5:** Association between subgastrocnemius bursitis, popliteal cysts and WOMAC measures

	Multivariable*			**Multivariable**^**#**^			**Multivariable**^**†**^		
	**Odds ratio**	**95% CI**	***P***	**Odds ratio**	**95% CI**	***P***	**Odds ratio**	**95% CI**	***P***
Total pain									
Subgastrocnemius bursitis	1.31	(0.99, 1.73)	0.05	1.31	(0.99, 1.73)	0.05	1.20	(0.91, 1.61)	0.19
Popliteal cyst	1.24	(0.98, 1.56)	0.07	1.24	(0.98, 1.56)	0.07	1.19	(0.94, 1.51)	0.15
Weight-bearing pain									
Subgastrocnemius bursitis	**1.34**	**(1.02, 1.77)**	**0.03**	**1.34**	**(1.02, 1.77)**	**0.04**	1.24	(0.94, 1.64)	0.14
Popliteal cyst	**1.27**	**(1.01, 1.60)**	**0.04**	**1.27**	**(1.01, 1.60)**	**0.04**	1.23	(0.97, 1.56)	0.09
Non-weight bearing pain									
Subgastrocnemius bursitis	0.85	(0.63, 1.13)	0.27	0.85	(0.64, 1.14)	0.28	0.85	(0.64, 1.14)	0.28
Popliteal cyst	0.97	(0.76, 1.24)	0.82	0.97	(0.76, 1.24)	0.82	0.97	(0.76, 1.24)	0.81
Stiffness									
Subgastrocnemius bursitis	**1.35**	**(1.03, 1.79)**	**0.03**	**1.36**	**(1.03, 1.80)**	**0.03**	1.29	(0.98, 1.72)	0.07
Popliteal cyst	1.20	(0.95, 1.52)	0.12	1.20	(0.94, 1.52)	0.12	1.18	(0.93, 1.49)	0.18
Dysfunction									
Subgastrocnemius bursitis	1.18	(0.9, 1.57)	0.23	1.18	(0.9, 1.57)	0.23	1.14	(0.85, 1.53)	0.37
Popliteal cyst	**1.29**	**(1.00, 1.67)**	**0.04**	**1.29**	**(1.00, 1.67)**	**0.04**	1.24	(0.96, 1.61)	0.09

Dependent variable: presence of pain, stiffness or dysfunction (yes versus no). Independent variable: subgastrocnemius bursitis or popliteal cyst, per grade. *Adjusted for age, sex, body mass index, disease status, and radiographic osteoarthritis; ^#^further adjusted for effusion; ^†^further adjusted for bone mass lesions and cartilage defects. Data in bold denote statistically significant results. WOMAC, Western Ontario McMaster Universities osteoarthritis index.

There were no significant interactions between sex and cystic lesions on OA measures so male and female subjects were combined for analysis (data not shown). The results remained unchanged when subjects with RA were excluded from analysis (data not shown).

## Discussion

To the best of our knowledge this is the first study to comprehensively examine the relationship between popliteal cyst, subgastocnemius bursitis and imaging abnormalities of knee OA as well as symptoms in older adults. We documented that popliteal cyst or subgastocnemius bursitis was not only associated with radiographic features (osteophytes and JSN), cartilage defects and BMLs, but also with knee OA symptoms, including weight-bearing pain, stiffness and dysfunction. The associations were independent of confounders including age, sex, BMI, radiographic OA and/or disease status, suggesting popliteal cyst and subgastrocnemius bursitis may play roles in the pathological process of OA.

In adults, the aetiology of popliteal cyst may be related to an inflammatory process, meniscal tears, or mechanical intra-articular derangements of the knee joint. In our study, knee surgical history was associated with popliteal cyst. We did not find that age, sex, BMI and smoking, the OA risk factors, were associated with popliteal cyst and subgastocnemius bursitis in older adults.

Both JSN and osteophytes are the key elements of radiographic OA. We documented that popliteal cyst was associated with osteophytes both in the medial and lateral tibiofemoral compartments whereas subgastrocnemius bursitis was associated with osteophytes and JSN in the medial tibiofemoral compartment, suggesting that popliteal cyst and subgastocnemius bursitis can co-exist with radiographic OA.

Nevertheless, radiographic JSN is a surrogate method used to assess knee cartilage, [18] and has been regarded as insensitive to change. Using arthroscopy, previous studies reported that 41% to 83% of joint disorders including meniscal tears and chondral lesions were associated with popliteal cysts [19-21]. MRI allows non-invasive, direct, accurate and reliable assessment of cartilage and other joint structures with their changes over time [22]. So far, there are no reports describing the associations between knee cystic lesions and MRI-based measurements such as cartilage defects and BMLs. Cartilage defects have been associated with tibiofemoral osteophytes, increased tibial bone area, decreased knee cartilage volume and type II collagen breakdown [13]. Knee cartilage defects are predictive of knee cartilage loss over 2 years, [23] suggesting a potential target for intervention in OA [24]. BMLs are commonly detected in knee OA as increased signal intensity within the bone marrow on MRI. BMLs are strongly associated with knee pain and have appeared to be a promising target for OA [25,26]. Using MRI we have reported that in the current study, popliteal cyst and subgastocnemius bursitis were positively associated with cartilage defects in all compartments. These associations were independent of covariates including female sex, age, BMI and radiographic features, all which have been associated with change in knee cartilage defects [27]. Furthermore, we found that both popliteal cyst and subgastocnemius bursitis were associated with medial tibiofermoal BMLs in older adults.

The reasons underlying the association between popliteal cyst, subgastocnemius bursitis and knee structural abnormalities, particularly in the medial compartment, are unclear. The medial compartment of the knee bears the majority of the mechanical loads. Abnormal loading can lead to changes in the composition, structures, [28] and mechanical properties of articular cartilage [29]. It is highly possible that weight-bearing loads can be perceived by joint fluid triggering two-way flow between the cavity and bursae, and thus induces more cartilage loss, BMLs or osteophyte formation in the medial compartment. It is also possible that there are elevated cytokines (for example, IL-6, TNF-α) or growth factors (for example, transforming growth factor (TGF)-β) in the cysts that can induce increased JSN, cartilage loss [30] and osteophytes [31].

Hill and colleagues reported 43.2% prevalence of popliteal cysts in knees with moderate or larger effusions, compared with 22.7% in those with little or no effusion, and the presence of popliteal cysts was not associated with knee pain [10]. Chatzopoulos *et al*. reported that popliteal cysts are a common finding in the knee among subjects with chronic OA pain [32]. These studies did not distinguish weight-bearing from non-weight-bearing pain, which are two distinct constructs of WOMAC pain [16]. By utilizing these classifications of pain, we have been able to detect that weight-bearing pain rather than non-weight-bearing pain was related to popliteal cyst or subgastocnemius bursitis. This finding is consistent with common belief, as weight-bearing pressure can cause compression of the adjacent cyst structures, and a relatively high proportion of weight-bearing pressure inside the cyst can induce knee pain. Increased pressure can promote extravasation of joint fluid through the posteromedial joint capsule into the bursae [33] and cause knee pain; however, it may not be related to joint cavity effusion observed simultaneously, because the significant association remained unchanged after further adjustment for effusion. The significant associations between knee pain and popliteal cyst and subgastocnemius bursitis may be mediated partly by cartilage defects and BMLs, because the associations decreased in magnitude and became non-significant after adjustment for these structural abnormalities. Although arthroscopic excision is commonly performed in symptomatic patients with popliteal cyst, our study suggests that such cysts and bursitis may not be the primary etiology for symptoms of OA, although this needs to be confirmed by future longitudinal studies.

The strengths of the present study lie in a large sample with knee radiographic and MRI measurements. There are a number of potential limitations in this study. First, this is a cross-sectional study, precluding our ability to draw conclusions about causal relationships between cysts or bursitis and the structural abnormalities. Nevertheless, the existence of the consistent relationships between cysts and many intra-articular pathologies of OA suggests that those cystic lesions may be involved in symptomatic and structural changes in OA. This is supported by a recent study, which confirmed that removal of the popliteal cyst and associated pathology would provide improvements in function and pain of the knee after surgery, and in patient satisfaction [34]. Second, we only assessed two cystic lesions in the current study. Other cystic lesions were not as prevalent as these two lesions [6] and low prevalence would disallow us to observe significant associations between other cystic lesions and outcome measures. Third, the response rate at baseline was 57%, possibly due to the demands on study participants that each visit took three hours. However, there were no significant differences in age and gender between those who responded and those who did not. Fourth, although we studied a well-characterized population of older adults with a high level of knee pain (46%), this randomly selected sample unavoidably included subjects with other diseases, which may have affected the associations. Nevertheless, the results were largely unchanged when the analysis was adjusted for disease status or the subjects with other diseases were excluded. Last, measurement error may influence results; however, given all measures (for example, cystic lesions, cartilage defect and BML) were highly reproducible, this is considered unlikely.

## Conclusions

This cross-sectional study documents that popliteal cyst and subgastrocnemius bursitis are associated with radiographic and MRI-detected joint structural abnormalities as well as knee symptoms. Longitudinal data will help resolve if they are a consequence or a cause of knee joint abnormalities.

## Abbreviations

BMI: body mass index; BML: bone marrow lesion; ICC: interclass correlation coefficient; IL: interleukin; JSN: joint space narrowing; MRI: magnetic resonance imaging; OA: osteoarthritis; OARSI: Osteoarthritis Research Society International; RA: rheumatoid arthritis; TASOAC: Tasmania Older Adult Cohort; TNF: tumour necrosis factor; WOMAC: Western Ontario McMaster Universities osteoarthritis index; WORMS: whole-organ magnetic resonance imaging score.

## Competing interests

The authors declare that they have no competing interests.

## Authors’ contributions

CD and YC conceived the study, CD, GJ and FC participated in its design and coordination, YC, WH, XW and BA performed MRI measurements. YC, GJ and CD drafted the manuscript. All authors revised the manuscript and gave final approval of the version to be submitted.
